# The Contribution of Historical and Morphological Studies on Herbarium Specimens to a Better Definition of *Chara pelosiana* Avetta (Charales, Charophyceae)

**DOI:** 10.3390/plants10112488

**Published:** 2021-11-17

**Authors:** Anna Millozza, Nadia Abdelahad

**Affiliations:** Dipartimento di Biologia Ambientale, Sapienza Università di Roma, 00185 Roma, Italy; anna.millozza@uniroma1.it

**Keywords:** charophytes, rice fields, morphology, haplostephanous species

## Abstract

The lectotype of Chara pelosiana Avetta 1898 was designated in 2000 by Langangen, who merged the species with *Chara fibrosa* Agardh ex Bruzelius. *Chara pelosiana* belongs to the section *Agardhia* Wood, but the true identity of the species has yet to be confirmed. The purpose of this work is to show some historical and morphological findings regarding this enigmatic species, on the basis of the analysis of herbarium specimens. The original material, which was studied by Avetta, is missing in Italian herbaria, but portions of it have been found in the Herbarium of Jena. Historical research on botanists related with this species resulted in the discovery of several specimens to be considered “original material”, and new unpublished localities in Northern Italy. Morphological observations have been made on portions of herbarium specimens as a contribution to unveil the taxonomic identity of this taxon. The specimens are diplostichous with ecorticate branchlets, have stipulodes in a single row, one or two per branchlet, and spine cell up to 1 mm long.

## 1. Introduction

*Chara pelosiana* was published in 1898 by Avetta [1] upon a specimen with a single row of stipulodes and ecorticate branchlets that was collected in 1886 in rice fields in S. Anna (near S. Cesario, Province of Modena, Northern Italy). *C. pelosiana* is one of the rarest haplostephanous species in Europe [1] (p. 229).

The species was part of Enrico Ferrari’s “small but interesting collection of Characeae” [1] (p. 230), collected for the University of Rome’s Botanical Institute, and was first studied by Alpinolo Pelosi, a young Natural Sciences student who died prematurely in 1887. Following Pelosi’s death, the Ferrari collection, as well as the few notes and observations left by Pelosi, were gathered by Carlo Avetta and stored in Parma [1] (p. 230). However, both the Rome and Parma Herbaria have since lost track of Ferrari’s collection and Pelosi’s documentation.

Pelosi identified the specimen as a variety of *Chara scoparia* Bauer (actually *Chara baueri* A. Braun) [1] (p. 234). When Avetta examined it, he noticed that the cortex was diplostichous rather than triplostichous, as it is in *C. baueri*, and assumed he was dealing with a new species [1] (p. 232). As a result, in honor of Pelosi, he named the species *Chara pelosiana.*

We started looking for Ferrari’s collection in 2009. A *C. pelosiana* specimen collected from S. Anna was discovered at the University of Turin Herbarium [2].

*C. pelosiana* has only been mentioned once in the Italian literature since Avetta’s publication [3] (p. 16). For nearly all of the twentieth century, there was no further record of the species in Italy. Plants that looked like *C. pelosiana* were discovered in 1999 in rice fields in the Province of Ferrara (Northern Italy) [4]. They were named *C. fibrosa* Agardh ex Bruzelius ssp. *benthamii* (A. Braun) Zaneveld, following Soulié-Märsche et al. [5].

It was Langangen who merged *C. pelosiana* with *C. fibrosa*, choosing fragments of the species collected in S. Anna and housed in the Herbarium of Oslo to be the lectotype of *C. pelosiana* [6]. In support of the merging of the two species, he cited Nordstedt, who identified the fragment kept at Olso as *C. gymnopitys* A. Braun or *C. flaccida* A. Braun, depending on the colour of the oospores. These two species (as well as a third one, *C. benthamii* A. Braun) were fused into *C. fibrosa* by Zaneveld [7] (p. 153).

Van Raam [8] named Avetta’s species *C. fibrosa* var. *pelosiana* (*nom. invalid.* according to [9]), and Krause [10], in agreement with Langangen, attributed *C. pelosiana* to *C. fibrosa* in a note at the end of his book. However, Wood [11,12] considered the species to be a form of *C. baueri.*

*C. fibrosa* is a species complex, which includes, in addition to the three species of Zaneveld mentioned above, other species, varieties, and forms merged by Wood [13,14,15].

The purpose of this work is to document historical and morphological findings on *C. pelosiana* herbarium specimens.

## 2. Materials and Methods

The historical search for *C. pelosiana* was based on an examination of the limited available literature [1,2,3] and, more importantly, a study of the herbarium materials kept in JE, LD, MOD, PAD, PARMA, PAV, RO, and TO (herbarium acronyms according to [16]). All of these Herbaria are related to botanists who have investigated or collected this species (see Appendix A). Further requests were sent without success to the Italian Herbaria BOLO, CAT, FI, NAP, PAL, and PI, which preserve historical collections of algae.

Furthermore, manuscript documents found attached to the specimens, as well as a selection of letters kept in the Archive of Botanical Garden of the University of Padua [17] provided significant additional information.

For the morphological investigation, portions of *C. pelosiana* from the Herbaria of MOD, TO, and PAV, as well as from the specimens kept in PAD Herbarium, were taken and transferred to Rome for examination and photography. The fragments stored in JE were insufficient for portions to be removed for further study. Morphological observations were made using a Zeiss stereomicroscope equipped with a Leica DFC 42 digital camera. The material was photographed either dry or after being rehydrated and decalcified using a 1N hydrochloric acid solution.

## 3. Results

### 3.1. Historical Findings

Unfortunately, neither RO nor PARMA, where Carlo Avetta worked from 1893 until his retirement, kept the original collection. Nevertheless, *C. pelosiana* specimens from the original site and other Italian locations have been discovered in several Italian and foreign Herbaria. 

Based on the importance of the exsiccata, the *C. pelosiana* specimens were divided into three groups. The data labels for each specimen were faithfully returned and noted.

#### 3.1.1. Herbaria That Keep Original Material of the Name *Chara pelosiana*

**Figure 1:**
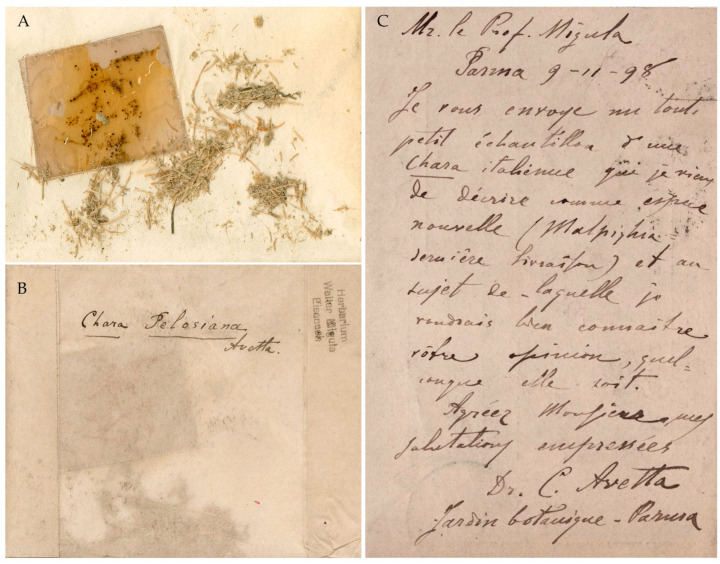
(**A**) JE fragments of *Chara pelosiana* from the missing RO specimen. (**B**) Avetta’s handwriting on the specimen envelope. (**C**) Postcard sent by Avetta to Migula on 9 November 1898.

**Jena Herbarium (JE)**: Small fragments of the species established by Avetta are kept in an envelope with the stamp “Herbarium Walter Migula Eisenach” in the top right corner. On the envelope, Avetta wrote “Chara Pelosiana Avetta” (Figure 1A,B).

A postcard from Avetta to Migula, dated November 9, 1898, is attached to the sample, and reports, in French, “Mr. le Prof. Migula | Parma 9-11-98 | Je vous envoye un tout petit échantillon d’une Chara italienne que je viens de décrire comme espèce nouvelle (Malpighia dernière livraison) et au sujet de laquelle je voudrais bien connaitre votre opinion, quelconque elle soit (…) Dr. C. Avetta | Jardin botanique—Parma” [(…) I am sending you a very small sample of an Italian *Chara* that I just described as a new species (Malpighia last issue) and would like to hear your opinion on it, whatever it is (…)] (Figure 1C).

Notes. The fragments are extremely significant because they come from the original material examined by Avetta, although the labels do not mention any locality. According to a footnote in Avetta’s paper, they were sent to Migula to obtain his opinion on the validity of the new species [1] (p. 229). In the same footnote, Avetta reports that the new taxon was published without confirmation from Migula, who was away for a few months and could not examine the specimen. As a result, the publication does not provide an illustration of the new species. Avetta goes on to say that the figures will appear in his next note on the Italian Characeae, which, however, was never published.

**Figure 2:**
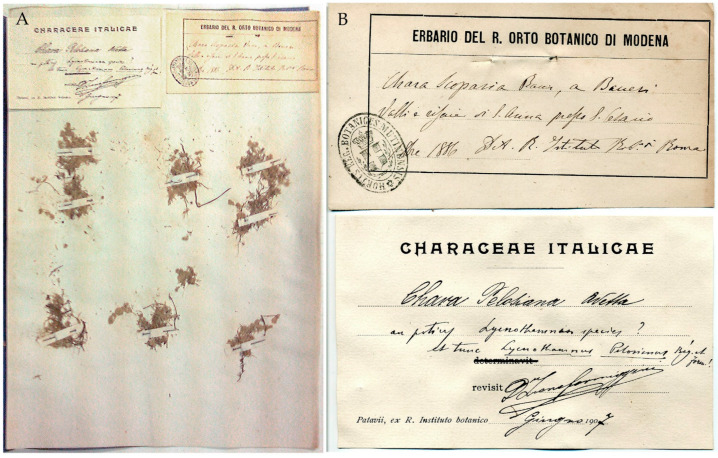
(**A**) MOD specimens of *Chara pelosiana* from the first duplicate of Ferrari’s original collection. (**B**) MOD preprint label with unknown handwriting (above) and Formiggini and Béguinot’s preprint revision label (below).

**Modena Herbarium (MOD)**: The specimen consists of six small fragments pinned to a herbarium sheet (Figure 2A). A preprint label from the Herbarium of Modena with the institution’s rubber stamps, “Hortus Reg. Botanicus Mutinensis”, provides the following information: “Chara Scoparia Bauer, a Baueri | Valli e risaie di S. Anna presso S. Cesario | 1 8bre [October] 1886” [unknown person *scripsit*] Det. R. Istituto Bot.^o^ [Botanico] di Roma” [a second, unknown person *scripsit*] (Figure 2B).

The preprint revision label was written by Leone Formiggini, who was engaged in revisionary work on the Italian Characeae with Augusto Béguinot (see Appendix A). The label is free within the folder, with the name of the research project, *Characeae Italicae*, at the top and the institution where the project was located, *Patavii, ex R. Instituto botanico*, at the bottom. The revision label bears the information “Chara Pelosiana Avetta *revisit* D^r^ Leone Formiggini Giugno [June] 1907” [Formiggini *scripsit*]. Béguinot added the comment, “an potius Lycnothamnus species? et tunc Lycnothamnus Pelosianus Bég. et Form.!” (Figure 2B).

Notes. The first duplicate of Ferrari’s original set collected in rice fields in the province of Modena is kept in MOD. When the Botanical Institute of Rome requested a collection of Characeae from this area, Ferrari was still working at the University of Modena (see Appendix A). Unfortunately, no Ferrari autograph labels can be seen in MOD. 

The Ferrari Characeae collection consists of 26 specimens of different species collected near Modena between 1878 and 1886, 14 of which were collected in 1886 (Table 1). All specimens have Modena Herbarium preprint labels with the stamp “Hortus Reg. Botanicus Mutinensis”. The collection is not numbered, there is no indication of *Legit*, and all of the labels were handwritten by two unidentified people. The homogeneity of the compilation becomes apparent when comparing the first handwriting, which included the binomial, locality, and date, as if the labels were filled in all at once by an amanuensis, rather than by a botanist. The second anonymous handwriting only provided information about who made identification, in this case, an Institute, the Botanical Institute of Rome.

Finally, it should be noted that Formiggini and Béguinot disagreed regarding the correct position of *C. pelosiana*, which, according to Béguinot, could be *Lychnothamus pelosianus* (see also below, the letter from Formiggini to Migula kept at JE, and the discussion).

**Figure 3:**
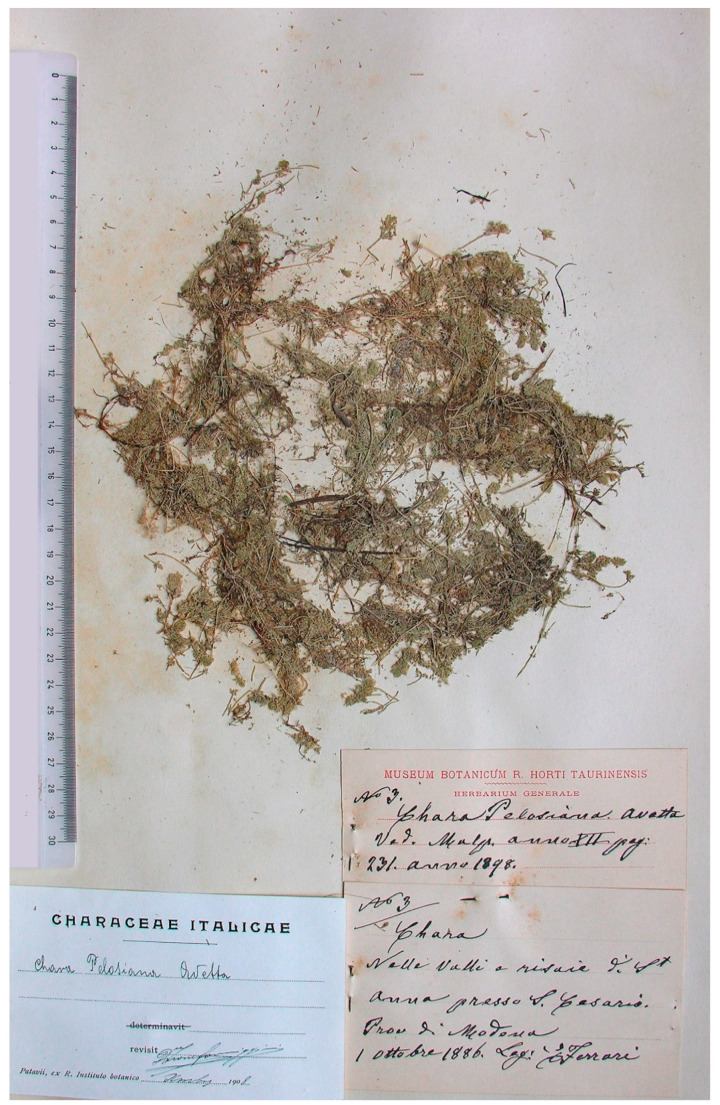
TO specimen of *Chara pelosiana* (No. 3) from the second duplicate of Ferrari’s original collection. Ferrari’s two handwritten labels (on the right) and Formiggini’s revision label (on the left).

**Turin Herbarium (TO)**: The specimen, which is nearly entirely fragmented, was found free within a folder with three labels. The first two labels, which are pinned to the herbarium sheet and almost joined by a third pin to form a single label, were handwritten by Ferrari (Figure 3. The first provides information about the specimen: “N° 3 | Chara | Nelle valli e risaie di St Anna presso S. Cesario. | Prov di Modena | 1 Ottobre 1886. Leg: E Ferrari”. The second reports the new binomial and references Avetta’s publication on a printed label from the Herbarium of Turin: “N° 3 Chara Pelosiana Avetta | Ved. Malp. anno XII pag: 231. anno 1898”. The third label, which was found free within the folder, is Formiggini’s preprint revision label: “Chara Pelosiana Avetta, *revisit* D^r^ Leone Formiggini, Xmbre [December] 1908”.

Notes. A second duplicate of the original set collected by Ferrari from rice fields in the province of Modena in 1886 is housed in TO, where Ferrari became the Curator of the Herbarium in November 1887 (see Appendix A). There are 25 Characeae specimens in the collection, 24 of which are numbered (Table 2). TO contains 19 additional Characeae specimens that were collected by Ferrari between 1887 and 1905, mainly from Piedmont and Valle d’Aosta.

The *C. pelosiana* specimen kept in TO is particularly valuable because the labels were handwritten by Ferrari, the original collector, and refer to both specimen No. 3 and the type locality, S. Anna [1] (p. 234).

TO also preserves two further specimens collected by Ferrari at S. Anna on September 19, 1899 (identified by Formiggini as *C. foetida* A. Br. and *C. fragilis* Desv. f. *subinermis* β *Hedwigii* Ag.), one year after Avetta’s publication and thirteen years after the first collection, suggesting that Ferrari tried unsuccessfully to again find *C. pelosiana*.

**Oslo Herbarium (O)**: The lectotype of *C. pelosiana* was designated by Langangen and is kept in the Oslo Herbarium [6]. The specimen, which is kept in an envelope, was identified by Otto Nordstedt. The original label reports, “Chara Pelosiana Avetta. | Valli e risaie di S. Anna presso S. Cesario Prov. di | Modena 18 1/10 86 [October,1 1886] | Leg. E. Ferrari. | Italien” [unknown person *scripsit*].

**Figure 4:**
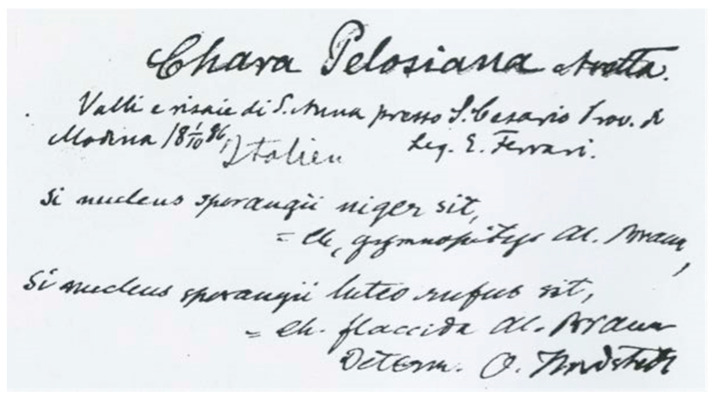
Label of the Oslo lectotype of *Chara pelosiana* with Nordstedt’s observation; from [6].

On the same label, Nordstedt made the following observation: “Si nucleus sporangii niger sit, | = Ch. gymnopitys Al. Braun, | Si nucleus sporangii luteo rufus sit, | = Ch. flaccida Al. Braun | Determ. O. Nordstedt” (lectotype) (Figure 4).

Notes. Langangen did not mention how the *C. pelosiana* specimen reached the Oslo Herbarium. He only recalled that Nordstedt, a charophyte authority at the time, was in close contact with the phycologist N. Wille from Kristiania (Oslo).

We also searched for *C. pelosiana* in the Lund Herbarium (LD), which houses the original Nordstedt herbarium, but found nothing.

#### 3.1.2. Herbaria That Keep Specimens of *C. pelosiana* Collected from New Localities

**Figure 5:**
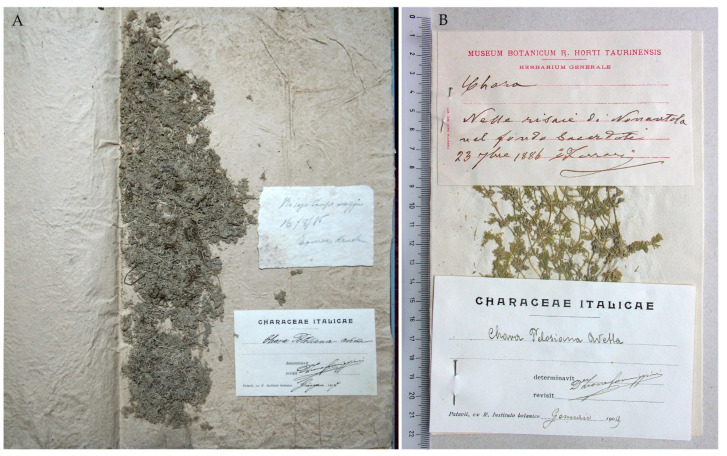
Unpublished specimens of *Chara pelosiana*. (**A**) PAV specimen collected by Traverso and Kruch from Campo Maggiore (Province of Pavia) in August 1886. Below is Formiggini’s revision label (**B**) TO specimen collected by Ferrari from Nonantola in September 1886 with Ferrari’s label (above) and Formiggini’s revision label (below).

**Pavia Herbarium (PAV)**: A large amount of material is kept free in a folder with a free label written in pencil: “Risaje Campo maggiore | 16/8/86 | Traverso e Kruch” [unknown person *scripsit*] (Figure 5A). Within the folder, there is also a free preprint revision label reporting, “Chara Pelosiana Avetta, *determinavit* D^r^ Leone Formiggini, Giugno [June] 1907”. [Formiggini *scripsit*] (Figure 5A).

Notes. This is the first unpublished specimen of *C. pelosiana* from a new station, the rice fields of Pavia Province (Campo Maggiore), which is about 150 km from the type locality (S. Anna). Giacomo Traverso and Osvaldo Kruch (see Appendix A) collected the specimen on August 16, 1886, a month and a half before the specimen from S. Anna was collected.

**Turin Herbarium (TO)**: A second specimen of *C. pelosiana* is mounted on an herbarium sheet with a printed label from the Herbarium of Turin pinned to the sheet reporting, “Chara | Nelle risaie di Nonantola | nel fondo Sacerdoti | 23 7bre [September] 1886, E Ferrari” [Ferrari *scripsit*] (Figure 5B). Formiggini’s preprint label is pinned to the sheet as well: “Chara Pelosiana Avetta, *determinavit* D^r^ Leone Formiggini, Gennaio [January] 1909”. [Formiggini *scripsit*] (Figure 5B).

Notes. This is the second unpublished specimen of *C. pelosiana*. It was collected by Ferrari from the same area in the province of Modena, the Nonantola rice fields, a week before the specimen of S. Anna was collected.

#### 3.1.3. Herbaria That Keep Portions of Specimens of *C. pelosiana* Removed from MOD and PAV

**Figure 6:**
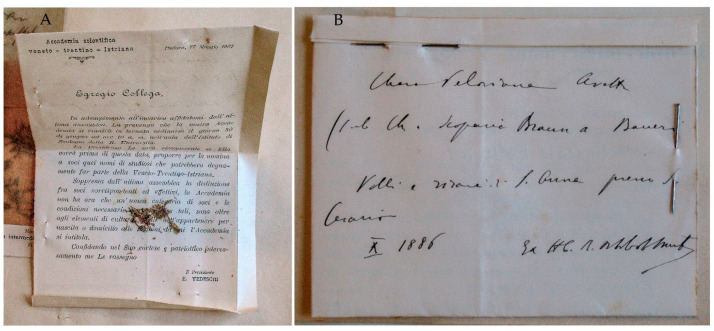
(**A**) PAD portion of *Chara pelosiana* removed from MOD specimen. (**B**) Béguinot’s indications on the envelope.

**Padua Herbarium (PAD)**: There are only a few MOD fragments (Figure 6A), which are kept in a recycled paper envelope with the indications written directly on it: “Chara Pelosiana Avetta | (sub Ch. Scoparia Braun a Baueri (sic) | Valli e risaie di S. Anna presso S. Cesario | X 1886 | Ex Hb. R. Ortobot.Mut(inensis) [Ex Herbario Regius Hortus Botani-cus Mutinensis]” [Béguinot *scripsit*] (Figure 6B).

**Figure 7:**
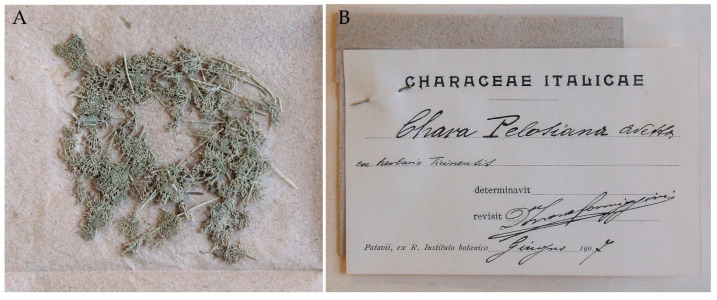
(**A**) PAD portion of *Chara pelosiana* removed from PAV specimen. (**B**) Formiggini’s revision label.

Instead, there are numerous fragments from PAV (Figure 7A). They are kept in an envelope with Formiggini’s preprint label pinned to it: “Chara Pelosiana Avetta | ex herbario Ticinensis | *determinavit* D^r^ Leone Formiggini Giugno [June] 1907” (Figure 7B).

Notes. The information on the two envelopes indicates that Béguinot and Formiggini, who both worked at Padua, took samples from MOD and PAV to their Botanical Institute for further investigation.

The portion from PAV specimen was especially valuable in our search for *C. pelosiana* specimens.

**Jena Herbarium (JE)**: In addition to fragments sent by Avetta, the Herbarium of Jena also conserves portions of specimens from MOD and PAV Herbaria.

**Figure 8:**
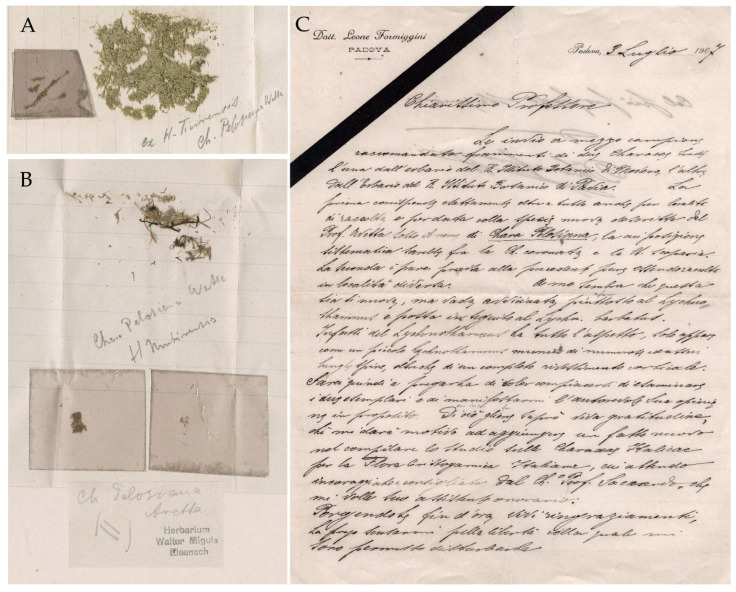
JE portions of *Chara pelosiana* sent by Formiggini to Migula in 1907. (**A**) Portions of PAV specimen with Formiggini’s handwritten notes. (**B**) Portions of MOD specimen with Formiggini’s handwritten notes (above) and Migula’s handwriting (below). (**C**) First page of Formiggini’s letter to Migula, dated 9 July 1907.

MOD’s portion consists of a few fragments, which are kept in a small envelope with the following indications written in pencil inside: “Chara Pelosiana Avetta | H Mutinensis” [Formiggini *scripsit*]. The revision by Migula is written in pencil on a label glued to the envelope reporting “II Ch. Pelosiana Avetta“with the stamp “Herbarium Walter Migula Eisenach” (Figure 8B).

PAV portion consists of several fragments, which are kept in a bigger envelope with the indications written in pencil inside as well: “ex H. Ticinensis | Ch. Pelosiana Avetta” [Formiggini *scripsit*] (Figure 8A). Migula’s revision is also written in pencil outside the envelope. It reports: “Ch. Pelosiana Avetta“with the stamp “Herbarium Walter Migula Eisenach”.

A letter from Leone Formiggini to Walter Migula, still in its original envelope, is attached to the specimens (Figure 8C). The letter is written in Italian and dated 9 July 1907: 

“Le invio (…) frammenti di due Caracee tratte l’una dall’erbario del R. Istituto Botanico di Modena, l’altra dall’erbario del R. Istituto Botanico di Pavia. La prima corrisponde esattamente oltre a tutto anche per località di raccolta e per data colla specie nuova descritta dal Prof. Avetta sotto il nome di *Chara Pelosiana*, la cui posizione sistematica sarebbe fra la Ch. Coronata e la Ch. Scoparia. La seconda è pure precisa alla precedente pure essendo raccolta in località diversa. A me sembra che questa sia sì nuova, ma vada avvicinata piuttosto al Lychnothamnus e posta in seguito al Lychn. barbatus. Infatti del Lychnothamnus ha tutto l’aspetto, solo appare come un piccolo Lychnothamnus munito di numerose ed assai lunghe spine, oltre che di un completo rivestimento corticale. (…)” “[I am sending you (…) fragments of two Italian Characeae, one from the Herbarium of the Royal Botanical Institute of Modena and the other from the Herbarium of the Royal Botanical Institute of Pavia. The first corresponds exactly, for locality of collection and date, to the new species described by Prof. Avetta under the name *Chara Pelosiana*, and its systematic position would be between *Ch. Coronata* and *Ch. Scoparia*. The second is very similar to the previous one even though it is collected in a different locality. It seems to me that this is indeed new, but it should be approached rather to *Lychnothamnus* and placed after to the *Lychn. barbatus*. In fact, it has in all the appearance of a *Lychnothamnus*, but it appears as a small Lychnothamnus equipped with numerous and very long spines, as well as a complete cortical covering. (…)].

Notes. According to the documentation, Formiggini submitted fragments of the two *C. pelosiana* specimens kept in PAD, which came from the portions removed from MOD and PAV, to Migula.

### 3.2. Morphological Findings

Measurements of the axes and lengths of the stipulodes were taken on the dry samples. They are summarized in Table 3 and Table 4.

These tables show that there are no consistent differences between the herbarium samples. Their axes have similar minimum and maximum diameters. The stipulodes’ lengths follow the same pattern. Additionally, preliminary phylogenetic analyses of partial chloroplast gene sequence data from the Nonantola and S. Anna collections stored in TO supports their con-specificity (Kenneth G. Karol, pers. comm.).

**Figure 9:**
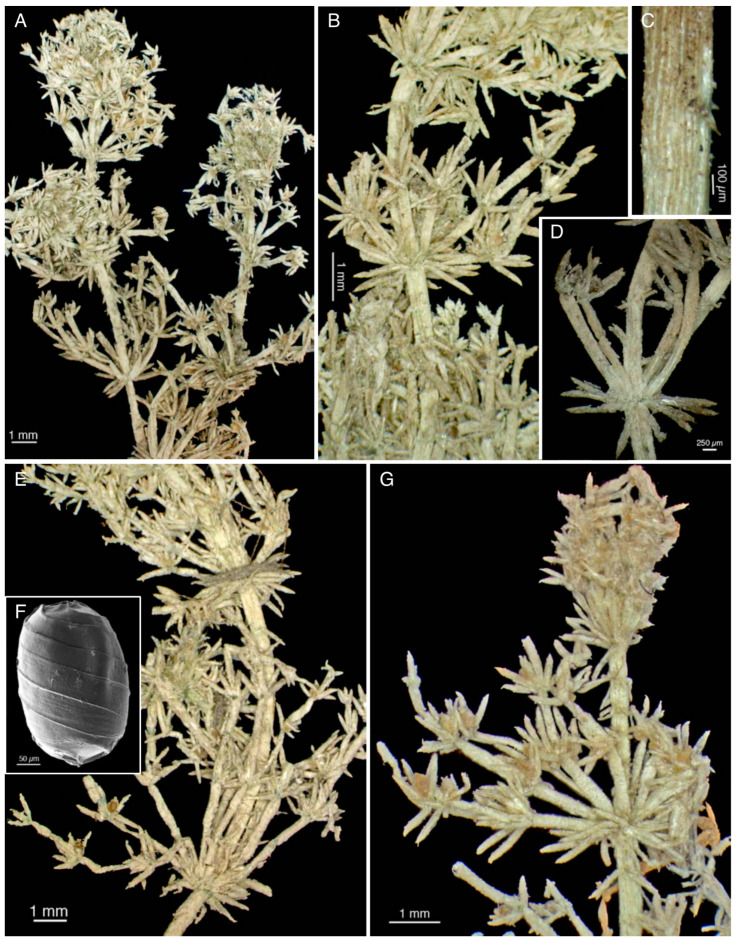
Morphologies of the portions removed from the specimens collected by Ferrari from S. Anna. (1 October 1886). (**A**–**D**) From the MOD specimen; (**C**) Details of the cortex; (**D**) Details of the stipulodes; (**E**,**F**) From the TO specimen (N. 3); (**G**) From the PAD portion.

**Figure 10:**
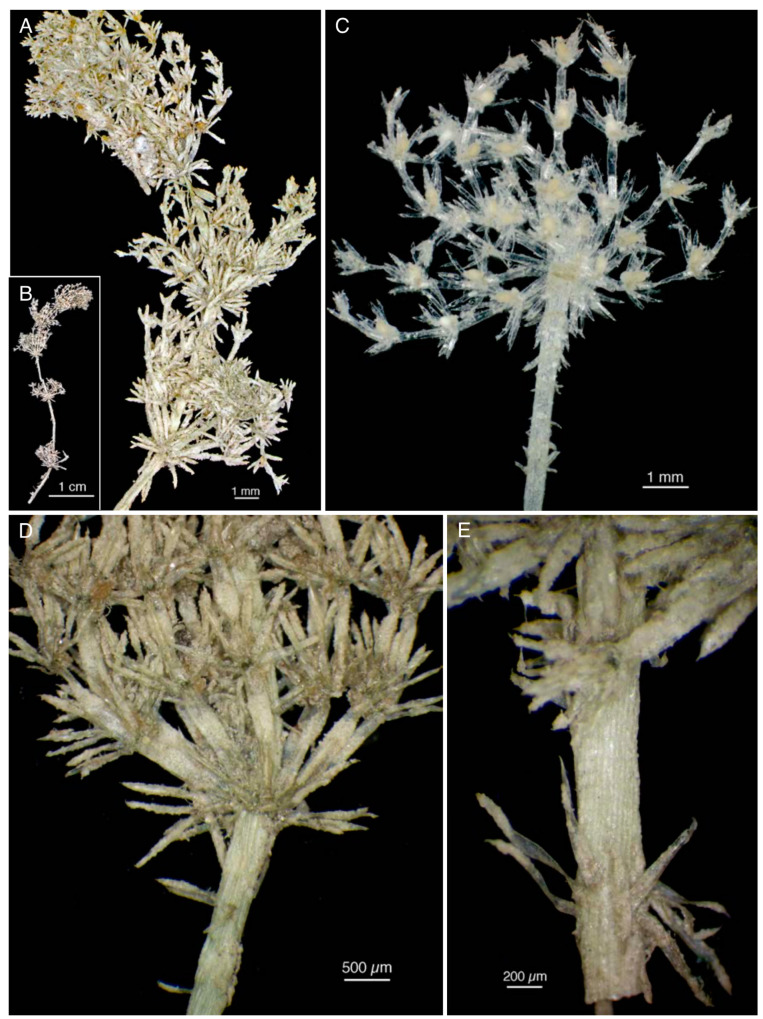
Morphologies of the portions removed from unpublished specimens. (**A**,**B**) From the TO specimen collected by Ferrari from Nonantola (23 September 1886); (**C**–**E**) From the PAV specimen collected by Traverso and Kruck from Campo Maggiore (16 August 1886); (**C**) Decalcified portion.

A stereomicroscope examination of these samples also revealed that they are morphologically comparable (Figure 9A–G and Figure 10A–E). Therefore, a single description can be extended to all the material removed.

The specimens, which are more or less heavily calcified, are almost all fragmented. Despite this, they do not appear to be longer than 7–8 cm (Figure 10B), as reported by Avetta [1] (p. 231), who observed them closer to the time of collection than we did. The axis diameter has mean values from 383 to 514 µm (Table 3). All the axes are corticated, diplostichous, isostichous or slightly tylacanthous (Figure 9C and Figure 10D,E), and bear spine cells (Table 5) generally longer than the axis, sometimes in a whorl (Figure 10E) and sometimes curved towards the axis (Figure 9A). Stipulodes are in a single row, perpendicular to the axis, one or two per branchlet, long to 1350 µm (Figure 9D,G and Figure 10A,D). Branchlets are (6)8-9(10) per whorl, totally ecorticate (Figure 10C), wide approximately half of the axes. They are composed of 3-4(6) segments bearing at each node, including the apical nodes, a crown of long bract cells (Figure 9G and Figure 10C). The two bracteoles are longer than the oogonia (Figure 9G). The mean length of the basal branchlet cells is 1.7–2.7 mm in the apical parts of MOD, TO, PAD, and PAV dry samples, and 3.5 mm in the fourth whorl of branchlets of the removed sample collected from S. Anna (TO).

The plants are monoecious with conjoined gametangia. The oogonia are 425–450 µm long (excluding the coronula) and 312–340 µm wide. The oospores are golden-brown, and have, in the dry material collected from S. Anna (TO), *c.* 350 µm in length, and 235 µm in width (Figure 9F). The spiral turns are 7–8. The antheridia in the portions removed from the PAV specimen are 250–270 µm in diameter.

In light of the information presented in Table 1 and Table 2, and considering the nomenclatural changes, the *C. pelosiana* species found in rice fields in the Modena area in the week of 23 September to 1 October 1886 were accompanied by: *C. vulgaris* L., *C. hispida* sensu auct. nonnull., *C. globularis* Thuiller, *C. tomentosa* L., and *Nitellopsis obtusa* (Desv.) J. Groves.

## 4. Discussion

### 4.1. The Studies of C. pelosiana by Pelosi, Avetta, Formiggini, and Béguinot

Letters from the scientific correspondence received by Pier Andrea Saccardo (1845–1920), a professor of botany and prefect of the Botanical Garden of Padua [17], help to explain the interests of the Rome Botanical Institute in Italian Characeae.

In a letter dated 4 November 1886, Pietro Romualdo Pirotta, the director of the Botanical Institute of Rome, requested a loan of Characeae specimens from the Padua Herbarium. The loan was for Alpinolo Pelosi, “a talented young student” who had been working with Characeae for a year and whom Pirotta encouraged to pursue a monographic study of the Italian species. Between the end of 1886 and the beginning of the next year, a great number of Italian specimens from most of the Italian Herbaria, including Ferrari’s small collection from the Modena area, were sent to Rome for this purpose [1] (p. 230) [17] (letters: 15 November 1886, 10 February 1887).

After Pelosi’s untimely death in August 1887, Carlo Avetta, who was Pirotta’s first assistant, was entrusted with the monograph of the Italian Characeae. Despite the difficulty presented by the large amount of material gathered in Rome and the study of a problematic group, Pirotta considered the work practically complete by the beginning of 1893 [17] (letters: 22 December 1890, 25 November 1891, 17 January 1893). However, Avetta had moved to Parma by the end of 1893, and Pirotta was forced to announce the conclusion of the study at the Botanical Institute of Rome, and he returned the loan of the Characeae of Padua [17] (letter: 12 April 1894).

Avetta was not a specialist of Characeae. The collections of the *General Herbarium* in RO house the only specimen of *Chara* collected by him. This specimen was identified by Formiggini and Béguinot (*Ch. crassicaulis*, Colli Astigiani, September 1886, det. Formiggini and Béguinot, *sine data*). Avetta’s revisions and determinations of the genera *Nitella*, *Tolypella*, *Lamprothamnium*, and *Lychnothamnus* can be found in the collections of the *General Herbarium* and *Cesati Herbarium* in RO, although they are nearly always unsigned.

Avetta confirmed [1] (p. 230) that his study of Italian Characeae began after Pelosi’s death (1887) while he was gathering the materials and notes left by the unlucky student as well as Ferrari’s collection. Both records have disappeared from Rome, but there is a record of a payment made to Ferrari in 1886 for his Characeae collection from the Modena area in the RO Archive [18].

Avetta resumed his study of Characeae in 1898, after a period of interruption [1] (p. 230), with the help of the regional collections kept at RO (*Roman Herbarium*). The Register of loans of RO shows a single loan of 44 specimens of Characeae sent to Avetta in Parma in 1898 [19]. The collection was returned to RO only ten years later, at the beginning of 1908, without any revisions. The sending took place a few months before Avetta’s publication on Malpighia, suggesting that Avetta still had Pelosi’s documentation and Ferrari’s collection with him when he left the University of Rome, perhaps as early as 1893, or that if these materials were forwarded to him later, they were sent privately.

Formiggini and Béguinot’s views on *C. pelosiana* can be deduced from specimens kept in TO, MOD, PAV, and JE Herbaria as well as from documentation in RO Archive. While Formiggini appears to have agreed on the validity of the new species (see revised labels from MOD and PAV, June 1907), Béguinot’s opinion was quite different (*an potius Lycnothamnus species? et tunc Lycnothamnus Pelosianus Bég. et Form.!*, MOD label), as he was in doubt as to whether *C. pelosiana* should be considered a new *Lychnothamnus* species. Nonetheless, in the letter sent to Migula (JE), Formiggini presented Béguinot’s doubts as his own, while both attached samples were sent with the binomial *C. pelosiana* written by Formiggini himself. It is unknown as to what Migula’s answer was, but the revisions on the specimens, handwritten by Migula, confirm the specimen was identified as *C. pelosiana*.

At the beginning of 1908, shortly after Avetta’s loan was returned, Formiggini and Béguinot examined the complete Characeae collection kept in RO, which consisted of 828 specimens from the three Herbaria: *Roman* (58), *General* (383), and *Cesati* (387). The Register of loans contains a detailed list of all species sent to them [20], revealing the absence of *C. pelosiana*, which was therefore no longer part of the RO collections ten years after Avetta’s publication.

Despite this, Formiggini considered still *C. pelosiana* to be valid at the beginning of 1909, as revealed by his subsequent revisions in TO: S. Anna (December 1908) and Nonantola (January 1909). Despite not having seen the original *C. pelosiana* material, he confirmed the validity of the new species by examining specimens in TO and MOD. Furthermore, he also recognized the unpublished specimens kept in TO and PAV as *C. pelosiana*. The one of the two new specimens discovered during our research is therefore the one collected by Ferrari from Nonantola (Modena) almost a week before the S. Anna specimen was collected. The other was collected by Traverso and Kruch near Pavia just over a month before the type specimen was found. This indicates two new Italian stations for this species’ distribution area.

### 4.2. The Double Numbering of C. pelosiana

According to Avetta’s paper [1] (pp. 234–235), it seems that two specimens were collected by Ferrari: “*Chara* N.° 3 della raccolta Ferrari” and “*Chara* N.° 101. Raccolta di Ferrari. (Nelle valli e risaie di S. Anna presso S Cesario, prov. di Modena, 1 ott. 86)”.

To bolster this impression, each of the two assumed *Chara* species were followed by different observations by Pelosi, which were fully published by Avetta [1] (pp. 234–235).

In the absence of the material seen by Avetta and Pelosi’s original notes, the examination of the specimens kept in TO and the information acquired in RO were decisive. 

TO preserves Ferrari’s only numbered collection, consisting of 25 specimens, 21 of which were collected between 20 September and 1 October 1886. This number appears to be correct, as Avetta, the last person to examine the Rome collection, described it as a “small collection” [1] (p. 230).

On the other hand, in TO, we recognized 14 of Pelosi’s revision labels, 12 of which are numbered (104, 107, 109, 111, 112, 113, 116, 118, 120, 122, 123, and 124).

In the collections of the *General Herbarium and Cesati Herbarium* in RO, Pelosi numbered his collections (4 out of 14), revisions (50 out of 55), and determinations (10 out of 15), but the numbering system, which ranges from 4 to 130, is seriously lacking and the numbers are frequently repeated.

Nonetheless, based on a comparison of Ferrari’s numbering in TO (up to 24) and Pelosi’s numbering in TO and RO (up to 130), it is almost certain that the original material of *C. pelosiana* should be regarded as a single gathering: Ferrari’s number 3 and Pelosi’s revision, identified by number 101.

### 4.3. Which Is the Correct Identity of Chara pelosiana?

Langangen [6] merged Avetta’s species with *Chara fibrosa*. Other authors have considered *C. pelosiana* to be *C. fibrosa* or a variety or form of this species [4,5,8,10].

*C. fibrosa* belongs to the section *Agardhia* Wood, which mainly includes exotic taxa (subsection *Agardhia*) and the European species *C. pelosiana* (subsection *Braunia*) [11,12].

*C. pelosiana* specimens found in herbaria or mentioned in literature in Italy [1,4] are all from rice fields.

In Zaneveld key [7], the essential differences between the three subspecies that this author includes in *C. fibrosa* are the colour of the ripe oospores (golden-brown in *C. fibrosa* ssp. *flaccida* and black in the other two: *C. fibrosa* ssp. *benthamii* and *C. fibrosa* ssp. *gymnopitys*) and the number of stipulodes (as numerous as the branchlets in *C. fibrosa* ssp. *benthamii*, twice as numerous as the branchlets in *C. fibrosa* ssp. *gymnopitys*).

In the examined specimens of *C. pelosiana*, the stipulodes were variable in number, sometimes nearly equal and sometimes more or even twice as numerous as the branchlets (Figure 9D,G and Figure 10A). Avetta reported that the number of stipulodes was equal to the number of the branchlets (10–12) [1] (pp. 232–233), while Langangen (despite having difficulty counting them) stated that there were 1–2 stipulodes per branchlet in the fragments of *C. pelosiana* that he observed in Oslo [6] (p. 250). It appears, therefore, the number of stipulodes in *C. pelosiana* is not constant, as has already observed by several authors in other species [7] (p. 154).

In contrast, the colour of the mature oospores in *C. pelosiana* was consistently found to be yellow-brown (Figure 9A,E).

Only one stipulode per branchlet is mentioned in the description of the type material of *C. fibrosa*, and its oospores are described as being “consistently a light golden-brown” [15]. Furthermore, *C. fibrosa* is endemic to the island of Guam (Micronesia) [15].

This investigation of *Chara fibrosa* led to the separation of two previously merged species in the section *Agardhia*, *C. fibrosa* and *C. wightii* (A. Braun) Casanova [15]. Thus, the correct identity of *C. pelosiana* cannot be determined until the section *Agardhia* will be fully revised. Meanwhile, in this work, we used the valid name *Chara pelosiana* established by Avetta [1].

## 5. Conclusions

This study of herbarium materials of *Chara pelosiana* revealed unexpected original material. Only the *C. pelosiana* specimen described by Avetta [1] is mentioned in the literature. Although Ferrari’s original collection is no longer kept in the Herbarium of Rome, two duplicates of the original set were discovered in the Modena and Turin Herbaria, each including a specimen of *Chara pelosiana* that can be considered “original material”.

Furthermore, two new additional localities were discovered in the Pavia and Turin Herbaria (rice fields of Campo Maggiore in the Province of Pavia and rice fields of Nonantola in the Province of Modena), providing new information about the Italian distribution area for this rare species.

This paper gives an example of how, in addition to traditional morphological, taxonomic, and systematic research rules, historical herbarium collections can be used to assist ecology, biogeography, and conservation biology research.

## Figures and Tables

**Table 1:** Duplicate of Ferrari’s Characeae collection from the Modena Herbarium (MOD). The specimens, collected in the Modena area in 1886, are listed in chronological order. For the abbreviations of names and authors, we reproduced the original labels. The *Chara pelosiana* specimen revised by Formiggini is marked in bold.

Date of Collection	Collection Locality	Determination According to the Botanical Institute of Rome	Revision of Leone Formiggini	Formiggini’s Revision Date
21 May	Castelvetro	*Chara foetida* A B. *longibracteata* A B *Laxior* A B	*Chara foetida* A. Br. f. *subinermes* β *longibracteata* A. Br.	May 1907
June	Rio di Valle Urbana	*Chara foetida* A B. *subinermis longibracteata*	*Chara foetida* A. Br. f. *subinermis* β *longibracteata* A. Br.	May 1907
21 September	Marshes at Villa S. Faustino	*Chara hispida* L. β *brachyphylla* A. B? (sic)	*Chara hispida* L. f. *macracantha* v. (sic)	May 1907
23 September	Nonantola, in the rice fields Sacerdoti	*Chara foetida* ABr. *subinermis* ABr. *longibracteata* ABr.	*Chara foetida* A. Br. f. *subinermis* ιι *typica* Mig. mixed with some fragments of *C. fragilis* Desv.	May 1907
23 September	Nonantola, in the rice fields Borsari	*Lychnothamnus stelliger* (Bauer) A. Br. var. *major* A. Br.	*Tolypellopsis stelligera* (Bauer) Migula v. *ulvoides* A. Br.	May 1907
23 September	Nonantola, in the rice fields Borsari	*Chara hispida* L *macrantha* A Br*. elongata* A Br.	*Chara hispida* L. f. *macracantha* α *typica*	May 1907
23 September	Nonantola, in the rice fields Sacerdoti along the ditch of the forest	*Lychnothamnus stelliger* (Bauer) A Br.	*Tolypellopsis stelligera* (Bauer) Migula v. *ulvoides* A. Br.	Apr 1907
26 September	Ditch above S. Marino near Carpi	*Chara hispida* L. sterile	*Chara hispida* L.	May 1907
26 September	Ditch above S. Marino near Carpi	*Chara foetida* ABr. *subinermis* ABr. *longibracteata* A.Br. *laxior* ABr.	*Chara foetida* A Br. f. *subinermis* β *longibracteata* ABr	May 1907
28 September	Rice fields Boretti at Villa S. Agnese	*Chara foetida* AB. *subinermis* ABr. *longibracteata* A.B. *laxior ABr.*	*Chara foetida* A Br. f. *subinermis* κ *clausa* A Br.	May 1907
28 September	Rice fields Boretti at Villa S. Agnese	*Chara foetida* AB. *brevibracteata* AB. *expansa* AB	*Chara foetida* A. Br. f. *paragymnophylla* δ *brevibracteata* Mig.	May 1907
1 October	Ditches between Castelfranco Emilia and Valli di St Anna	*Chara foetida* A. B. *subinermis—longibracteata laxior* AB.	*Chara foetida* ABr. f. *subinermis* β *longibracteata* ABr.	May 1907
1 October	Rice fields of S. Anna near S. Cesario	*Chara hispida* L *micrantha* AB. *microphylla* AB.	*Chara hispida* L. f. *micrantha* π *brachyphylla*	May 1907
1 October	**In the valleys and rice fields of S. Anna near** **S. Cesario**	***Chara Scoparia* Bauer, a *Baueri***	*Chara pelosiana* Avetta *	**June 1907**

* an potius *Lycnothamnus* species? et tunc *Lycnothamnus Pelosianus* Bég. et Form.! [Beguinot’s revision on the same revision label, see Figure 2B].

**Table 2:** Duplicate of Ferrari’s Characeae collection from the Turin Herbarium (TO). The specimens, collected in the Modena area in 1886, are listed in chronological order. For the abbreviations of names and authors, we reproduced the original labels. The two specimens of *Chara pelosiana* revised by Formiggini are marked in bold.

Date of Collection	Collection Locality	Collecting Number	Determination of Ferrari	Determination/Revision/Confirmation of Formiggini	Formiggini’s Revision Date
22 April	In the ditches around Carpi	12	*Nitella capitata* Nees	*Nitella capitata* (N.ab. Es.) Ag.	December 1908
May	Villa Albareto ditches, site called “i Tagliati”	21	*Chara*	*Chara foetida* f. *subinermis* a *normalis* Mig.	January 1909
21 May	“Bosco Bontempelli” in ponds of water (Colli di Castelvetro)	24	*Chara foetida* A. Br *sub. var. longibracteata* A. B β *laxior* A. Br	*Vidit* = *Confirmavit*	-
June	Sassuolo: along Rio di Valle Urbana	23	*Chara foetida* A. Br *a subinermis. longibracteata* A. Br.	*Chara foetida* A.Br. *f. subinermis* β *longibracteata* A. Br.	June 1910
20 September	Nonantola, in the rice fields Borsari	20	*Chara hispida* L. *C. micrantha* A. Br *C. elongata* A. Br.	*-*	-
21 September	Marshes at S. Faustino	13	*Chara hispida* a*. brachyphylla* A. Br	*Chara hispida* L. f. *micracantha*—*brachyphylla* A.Br.	December 1908
21 September	Marshes at Villa S. Faustino	14	*Chara foetida* A. Br β *longibracteata* A. Br	*Vidit* = *Confirmavit*	-
21 September	Marshes at S. Faustino	15	*Chara foetida* A. Br	*Vidit* = *Confirmavit*	December 1908
**23 September**	**Nonantola, in the rice fields Sacerdoti**	**-**	***Chara***	***Chara pelosiana* Avetta**	**January 1909**
23 September	Nonantola, in the rice fields Sacerdoti	16	*Chara foetida* A. Br a *subinermis* β. *longibracteata* A. Br.	*Vidit* = *Confirmavit*	-
23 September	Nonantola, in the rice fields Borsari	17	*Lychnothamnus stelliger* A. Br. var. *major* A. Br.	*Tolypellopsis obtusa* (Desv.) Bèg. et Formigg. var. *ulvoides* (Bert.) Bèg. et Formigg.	December 1908
23 September	Nonantola: in the rice fields Sacerdoti along the ditch of the forest	18	*Chara*	*Chara fragilis* Desv. f. *microptila* β *Hedwigii* Ag.	January 1909
23 September	Nonantola, in the rice fields Sacerdoti	19	*Lychnothamnus stellinger* (sic) A. Br. var. *major* A. Br.	*Tolypellopsis obtusa* (Desv.) Bèg. et Formig. var. *ulvoides* (Bert.) Bèg. et Formigg.	December 1908
23 September	Nonantola: in the rice fields Sacerdoti along the ditch of the forest	22	*Chara*	*Chara foetida* A. Br. f. *subinermis* A. Br. *typica* Mig.	January 1909
26 September	Ditches above S. Marino near Carpi	10	*Chara hispida* L.	*Chara hispida* L. f. *microcantha* λ *vulgaris*	December 1908
26 September	Ditches above S. Marino near Carpi	11	*Chara foetida* A. Br β *longibracteata* A. Br. β *laxa*	*Vidit* = *Confirmavit*	-
28 September	Rice field fondo Borretta (sic) at Villa S. Agnese	7	*Chara foetida* A. Br β *longibracteata A. Br* β *laxa*	*Vidit* = *Confirmavit*	-
28 September	Rice fields fondo Borretti at Villa St Agnese	8	*Chara foetida* A. Br β: *longibracteata* A. Br. *expans.* A. Br	*Vidit* = *Confirmavit*	-
28 September	Rice fields fondo Borretti Villa S. Agnese	9	*Nitella tenuissima* Desv.	*Nitella tenuissima* (Desv.) Coss. et Germ. f. *major* Mig.	December 1908
1 October	Ditches between Castelfranco Emilia and Valli di St Anna	1	*Chara foetida* A. B. *|* *C. subinermis* A. Br. *|* *C. longibracteata* A. Br. *|* *C. laxior* A.Br	*Vidit = Confirmavit*	-
1 October	In the valleys and rice fields of S. Anna at San Cesario	2	*Chara*	*Chara hispida* L. f. *micrantha* π *brachyphylla*	January 1909
**1 October**	**In the valleys and rice fields of S. Anna near San Cesario**	**3**	***Chara |Chara pelosiana* Avetta Ved. Malp. anno XII. pag: 231. anno 1898.**	***Chara pelosiana* Avetta**	**December 1908**
1 October	In the valleys and rice fields of S. Anna near San Cesario	4	*Chara hispida* Thuil var. *microphylla* Schumach	-	-
1 October	In the valleys and rice fields of S. Anna near San Cesario	5	*Chara*	*Chara ceratophylla* Wallr.	January 1909
1 October	In the valleys and rice fields of S. Anna near San Cesario	6	*Chara hispida* L. var *microphylla* Schumach.	*-*	-

**Table 3:** Diameters of axes. All values are presented in µm.

Herbarium	Locality	Date of Collection	Collector	Minimum	Maximum	Mean	Number of Axes Measured
MOD	S. Anna	1 October 1886	Probably Ferrari	312	458	383	11
TO	S. Anna	1 October 1886	Ferrari	434	566	486	7
PAV	Campo Maggiore	16 August 1886	Traverso and Kruch	399	578	514	3
TO	Nonantola	23 September 1886	Ferrari	438	566	483	5
PAD	S. Anna	1 October 1886	Ferrari	325	469	401	5

**Table 4:** Lengths of stipulodes. All values are presented in µm.

Herbarium	Locality	Date of Collection	Collector	Minimum	Maximum	Mean	Number of Stipulodes Measured
MOD	S. Anna	1 October 1886	Probably Ferrari	965	1274	1151	4
TO	S. Anna	1 October 1886	Ferrari	1277	1470	1349	3
PAV	Campo Maggiore	16 August 1886	Traverso and Kruch	962	1614	1255	5
TO	Nonantola	23 September 1886	Ferrari	1265	1337	1301	2
PAD	S. Anna	1 October 1886	Ferrari	1194	1312	1253	2

**Table 5:** Lengths of spine cells. All values are presented in µm.

Herbarium	Localiy	Date of Collection	Collector	Minimum	Maximum	Mean	Number of Spine Cells Measured
MOD	S. Anna	1 October 1886	Probably Ferrari	301	638	456	6
TO	S. Anna	1 October 1886	Ferrari	397	807	557	4
PAD	S. Anna	1 October 1886	Ferrari	463	856	685	5
TO	Nonantola	23 September 1886	Ferrari	157	1.060	590	6
PAV	Campo Maggiore	16 August 1886	Traverso and Kruch	336	879	645	12

## Appendix A. Biographical Notes

The use of an exclamation point (!) indicates that personal data were retrieved from the Municipal Registry Offices. For bibliographical details see [21,22].

**Avetta, Carlo**

Turin, 13 March 1861–Turin, 10 March 1941

Botanist. Avetta’s academic career began at the University of Rome, where he worked as the *first assistant* to Pietro Romualdo Pirotta (1853–1936), the director of the Botanical Institute, from 1885 until 1893. His second and final position was as a professor and director of the Botanical Garden at the University of Parma (1893–1935). He was first interested in cryptogamic studies, especially fungi, and then in Scioa’s African collections, which he studied and illustrated. Anatomy and cytology were two of his most important subjects of study. In Parma, he was particularly interested in the diffusion of medicinal plant knowledge. From his study on Italian Characeae species, only his paper on *Chara pelosiana* remains. His announced second note on Italian Characeae was never published.

**Béguinot, Augusto**

Paliano (Frosinone), 17 October 1875–Genua, 3 January 1940

Botanist. Béguinot graduated with a degree in Natural Sciences from the University of Rome in 1898. His academic career covered six universities: Padua (1900–1921), where he worked as an assistant to Pier Andrea Saccardo; Ferrara (1918–1921); Sassari (1921), where he became professor; Messina (1922); Modena (1924–1929); and Genua (1929–1940). He published a large number of scientific works on flora, systematics, phytogeography, and the history of botany. His interest in the Characeae was restricted to the time he worked at the University of Padua with Formiggini (see below), and he published two contributions with him [23,24]. Their final paper on the Characeae of Italy was never published.

**Ferrari, Enrico**

Modena, 3 November 1845–Turin, 2 November 1921

Gardener at the Botanical Garden of Modena. Ferrari began studying plants in a self-taught manner and quickly advanced to become a skilled collector and an expert florist as well as an appreciated contributor to the *Flora del Modenese e del Reggiano* [25] (p. 5). According to the RO Archive (2), Pirotta was most likely the one who asked Ferrari for a collection of Characeae for Pelosi’s study in 1886. From 1887 to 1921, he was the Curator of the Turin Herbarium. Ferrari was the main author of the reorganization of Turin’s collections, while also contributing significantly to their increase in assiduous excursions, especially in Piedmont and the Valle d’Aosta.

**Formiggini, Leone**

Padua, 10 December 1879–Padua, 7 June 1963 (!)

Formiggini graduated from Padua on 5 December 1904 with a thesis titled “*Contribution to the knowledge of the Caracee from Padua*” [26]. From 1906 until 1909, he worked as an *honorary assistant* at the University of Padua’s Botanical Institute under the direction of Pier Andrea Saccardo (1845–1920) [27]. Formiggini was the author of the most important contribution, to the knowledge of the Italian Charophytes during this period, through his critical reviews of the Charophytes of Sicily [28], Veneto and Mantovano [29], and Lazio [30]. In collaboration with A. Béguinot, he became interested in some vicarious Characeae of the Italian Flora [23,24]. His collaboration with the Botanical Institute was interrupted in 1910, leaving the announced general work on Italian Charophytes unpublished [3]. However, his extensive and accurate work of revision and identification of Italian Charophytes *exsiccata* is still preserved in the Italy’s major public and private Herbaria. In a 1939 information-curriculum [31], Formiggini is still listed as a Padua resident, and his lone occupation appears to be that of covering various positions, some honorific, in Padua’s Societies, Academies, and Associations.

**Kruch, Osvaldo**

Pavia, 1 November 1864–Luino (Varese), 7 October 1942

Botanist. Kruch graduated with a degree in Natural Sciences from the University of Pavia on 29 June 1886 [32]. The following year, he conducted postgraduate studies at the Botanical Institute of the University of Rome under the direction of Pietro Romualdo Pirotta. In May 1893, he was named curator of the Rome Herbarium, and in January 1894 he was promoted to *first assistant*, covering the vacancy left by Carlo Avetta. Finally, between 1896 until 1935, he was a professor at the Agricultural Experiment Institute of Perugia (now Faculty of Agriculture). His studies were mostly focused on histology and plant anatomy.

**Migula, Walther**

Zyrowa, Upper Silesia (now in southern Poland), 4 November 1863–Eisenach (Germany), 23 June 1938

German botanist. Migula graduated from the University of Breslau (1888). He taught at the Karlsruhe Institute of Technology until 1895 and then at the Forestry School in Eisenach (1895–1915). His works on the cryptogamic flora, bacteriology, and plant physiology are well known. *Die Characeen Deutschlands, Oesterreichs und der Schweiz* in *Rabenhorst’s Kriptogamen-Flora* (1889/97) and *Synopsis Characearum europaearum* (1898) are considered fundamental works on the Characeae family. He published the *Characeae exsiccatae* (1892–1901) series with P. Sidow (1851–1925) and L.J. Wahlstedt (1836–1917), which includes 150 specimens in six fascicles.

**Pelosi, Alpinolo**

S. Pancrazio Parmense (Parma), 2 November 1865 (!)–Anguillara Sabazia (Rome), 1 August 1887 (!), not 1888 [1] (p. 230, note 1).

As a student of Natural Sciences at the University of Rome (1885–1887), he became a favorite pupil of Pietro Romualdo Pirotta, the director of the Botanical Institute. In 1885, he began studying the Italian Charophytes on the collections of the Herbarium of Rome and those of the main Italian Herbaria, under the direction of Pirotta (1). The next summer, Pelosi died in the waters of the Lake of Martignano (Rome), where he was collecting algae and other water plants on behalf of the Botanical Institute [33,34]. He was only 21 years old at the time. Many of his revisions, determinations, and observations on Italian Charophytes remain unsigned in the exsiccata of Rome and the main Italian Herbaria.

**Traverso, Giacomo**

Pegli (Genoa), 8 May 1849 (!)–?

From 1877 to 1909, Traverso was the head gardener at the Pavia Botanical Garden [35,36].

He was the father of Giovanni Battista Traverso (1878–1955), a mycologist, and Onorato Traverso (1881–1960), the head gardener of the Rome Botanical Garden.
